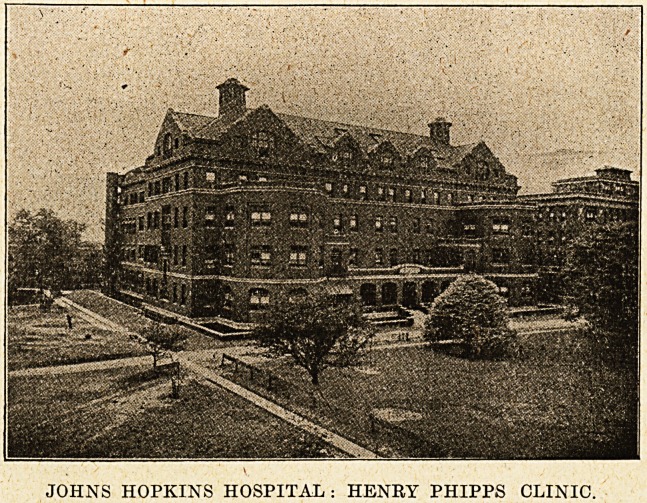# The Johns Hopkins Hospital, Baltimore

**Published:** 1917-01-27

**Authors:** 


### THE JOHNS HOPKINS HOSPITAL, BALTIMORE.

### UNIQUE, UP TO DATE, AND INSPIRING.

This hospital is unique in the circumstances of its original planning and creation, its splendid efficiency from its opening to the present time, and the object-lessons it affords to every visitor in proportion to the knowledge and experience of hospitals and the treatment of disease which each may possess. To be associated as a member with the staff of this hospital is to be penetrated with the highest standards of efficiency. We have felt often, and never more forcibly or certainly than during the visit of inspection we paid to this hospital on October 1, 1916, that no hospital administrator's or practitioner's education is complete until he has mastered the Johns Hopkins Hospital system and methods and fully absorbed their atmosphere. We have had the privilege of knowing those who have been successively responsible for the hospital's creation, development, and magnificent progress, and the opinion here expressed rests upon full knowledge and heartfelt conviction.

THE HARRIET LANE HOME: CHILDREN'S CLINIC.

Incorporated in 1867, it was not opened until 1889; the twenty-two years were fully utilised to develop the estate, to obtain precise and technically accurate knowledge of the best ideals and working models of hospitals, and to organise and equip the system of administration with the most up-to-date machinery and means. Those at the helm were fortunate in obtaining the best men to secure new departures in many departments. When first opened, this hospital created wide attention and interest. The work accomplished within its walls has included much which has led to important developments in the treatment of disease and the training of a number of the greatest members of the medical profession, who have attained high positions and possess a world-wide reputation. The best and nothing but the best possible has been aimed at and secured at Baltimore.

It was not possible at the outset to carry out the whole scheme or to create all the departments which were held to be essential to provide a centre of medical treatment and instruction of supreme value to the profession. Commencing with most of the departments to be met with in the best types of hospitals in 1889, the trustees have gradually extended the area of their work, and the latest additions include the Harriet Lane Home, a specially planned hospital for children; the Brady Urological Clinic; and the Henry Phipps Psychiatric Clinic. There are private rooms and suites of rooms ranging from $1 to $2 a day; the price depends upon the position of the patient and whether each is with or without exclusive bath privileges. The rates for the children's clinic range from 16c. to $2 a day. We give an illustration of the children's clinic, being the front view of the Harriet Lane Home for invalid children. There are also three low isolated pavilions: A for the care of diphtheria cases, B an observation ward where children are detained before admission to the general wards, C the scarlet-fever pavilion. We hope to publish plans and a fuller description of these buildings on a subsequent occasion. An interesting feature of the children's clinic is that mothers are allowed to accompany their children when they are admitted as patients. This feature has proved to be of the first importance. Many mothers whose little children require hospital treatment will not consent to be separated from them, and even if they finally consent they are most unhappy during the separation. It has, therefore, been found desirable to allow the mother to enter with the child, unless the condition of the child absolutely forbids this or the disease is of a contagious nature. In most instances the mother occupies a separate room and obtains her meals in the staff dining-room. It is the invariable practice of the managers to refuse to allow the nurses and maids engaged in the care of patients to occupy their time in serving meals to healthy people. Experience in the working of the Harriet Lane Home for Invalid Children has enabled the system of management to be perfected. It is a system that might be advantageously studied by the managers of many hospitals for children.

The admission of both free and part-pay patients to the Johns Hopkins Hospital is in the hands of the superintendent. The free beds are reserved for the sick poor of Baltimore and suburbs, and for accident cases from Baltimore and the State of Maryland. Other indigent patients pay a uniform rate of $2 per week until discharged. An operating-room fee of $1 is charged to surgical patients in the public wards. The hospital is designed for cases of acute disease. Cases of chronic disease are not admitted except temporarily.

### Its Atmosphere and Mental Clinics.

The whole atmosphere of the Johns Hopkins Hospital from the time the visitor enters the administration building to the time of his departure, after some hours of inspection, is one which elevates and inspires confidence. The hospital world contains nothing quite like it elsewhere, and it was cheering to note that in each new addition to the original buildings care has been taken to preserve and perpetuate the same atmosphere. The result rests in a measure upon the artistic sense and apprehension displayed by those responsible for the plans. Every section of the hospital has its own separate reception-room, and each reception-room is especially designed on a novel principle, distinct from every other. The impression given is that even the most timid of friends can properly leave the patient in the hospital, with assured confidence that everything possible will be done to expedite recovery and facilitate the interests and happiness of the patients within its walls.

The Henry Phipps Psychiatric Clinic is a wonderful creation in the architectural and artistic senses. Here the relatively small space of grass land which the site provided has yet yielded grounds for an institution of the highest rank. Someone with experience and great taste must have cooperated in arranging the grounds and gardens. Taken as a whole and in detail, the Henry Phipps Psychiatric Clinic may be regarded as a model, which we could wish to see reproduced, so that mental cases, in their early stages, may in increasing numbers be placed under the most favourable conditions to win them back to health, to secure the alleviation of their maladies, and not infrequently their complete recovery. The most recent addition is the Brady Urological Clinic. This clinic is planned for the reception of pay patients, and everything has been provided to simplify and promote the effective treatment of these sometimes difficult and trying cases, and to place them under hygienic and other conditions, frequently of novelty and always with attractive success.

JOHNS HOPKINS HOSPITAL: HENRY PHIPPS CLINIC.

### The Private Patient Service.

The rate for private rooms is from £1 to £2 per diem. All accounts are payable weekly in advance. This charge covers board, ordinary medicine, and the divided attention of the regular nurses on duty, which is all that is required, unless the patient is very ill or nervous. A charge of £2 is made for the use of the operating-room. X-ray examinations or treatment, massage, rare and expensive drugs if ordered for a specific case, champagnes, wines, and mineral waters entail an additional charge. The charge for the exclusive service of a graduate nurse, night or day, is £6 10s. per week. No graduate nurse is allowed to remain on duty for more than twelve hours or to sleep in the room with the patient. Persons entering the hospital as the private patient of any member of the staff are to arrange with the physician selected the amount of the fee for professional services. The fee to hospital cases for professional services, in addition to the daily rate for room and board, is made to suit the circumstances of each patient. When the occasion warrants it, the fee may be waived entirely. When the services of a specialist or of a dentist are required, it is customary for such specialists to render bills for their services. Visiting hours for patients, subject to the doctor's orders, are 10.30 A.M. to noon, 2 P.M. to 8.30 P.M. The hospital does not provide for personal laundry, and is not responsible for money or valuables brought by the patients unless deposited in the hospital safe.

No person employed or connected with the hospital is allowed to receive gratuities from a patient, unless with the consent of the executive committee or the superintendent. Operations are not performed before 8 A.M. or after 6 P.M., or on Sundays or holidays except in urgent cases. The whole of the arrangements throughout this service are attractive and good.

### The Medical Staff.

It may be of interest to state that each staff—medical, surgical, gynæcological, psychiatric—is under the immediate direction of the physician, surgeon, or medical practitioner-in-chief. The chiefs are paid a salary, half by the university and half by the hospital. Each chief has his house staff, consisting of the resident (who is also paid a salary by the hospital), assistant residents, and interns. The number of members on each staff varies with the amount of work done. Appointments are made for one year, and the residents are usually reappointed on the medical and surgical staffs, so that they may serve for two, three, or more years. In the meantime, most of the interns are graduates of the Johns Hopkins Medical School, and an intern appointed to one service serves his year's appointment on that service alone. The present resident staff, including three pathologists, consists of over fifty members. The largest, which is the medical staff, has a resident at its head, with six assistants and four interns. The medical student during the last two years of his training lives practically within the hospital, in close touch with the patients.

## Figures and Tables

**Figure f1:**
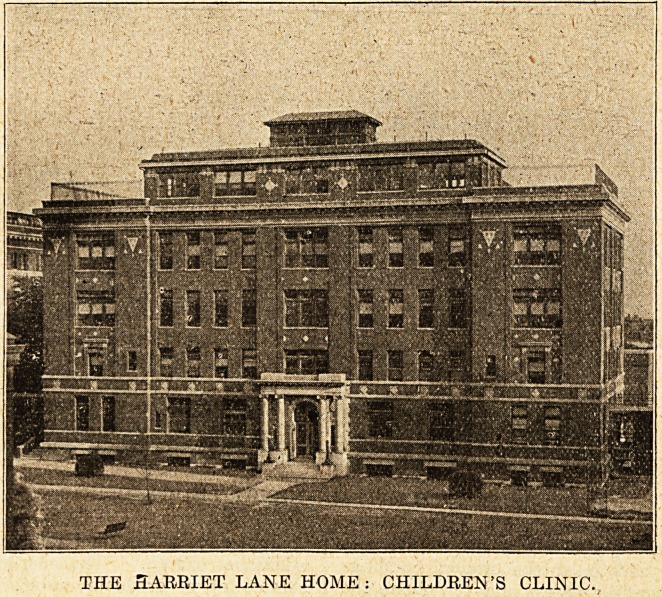


**Figure f2:**